# Myeloid related proteins 8 and 14 (mrp 8/14) - potential biomarkers of disease activity of arthritis in children with trisomy 21

**DOI:** 10.1186/1546-0096-12-S1-P182

**Published:** 2014-09-17

**Authors:** Charlene Foley, Emma MacDermott, Orla Killeen

**Affiliations:** 1 Rheumatology, National Centre for Paediatric Rheumatology (NCPR), Dublin, Ireland

## Introduction

JIA is an umbrella term used to describe a heterogeneous group of diseases. To date no specific markers exist in clinical practice to predict disease activity & outcome. MRP 8/14 are calcium-binding proteins secreted by infiltrating phagocytes in synovial inflammation. Studies have shown that serum (Se) concentrations correlate sensitively & specifically with synovial inflammation in JIA. It is believed that they are predictive biomarkers that can identify subclinical disease activity, patients at risk of relapse during times of clinically inactive disease, and patients more likely to respond to Rx with MTX. To date there have been no studies looking specifically at their use in DA. Research Q(s) - 1. Are MRP8/14 accurate markers of inflammation in DA? 2. Do MRP 8/14 levels in Se correlate to Synovial Fluid (SF)?

## Objectives

Evaluate the use of CRP, ESR & MRP 8/14 as biomarkers of disease activity in DA & JIA.

## Methods

Between May 2013-May 2014, new cases of JIA & DA attending the NCPR had blood drawn to measure their CRP, ESR & MRP 8/14 levels at dx. Corresponding AJC was documented. Paired SF samples were taken for analysis from children requiring steroid JIs as Rx for their arthritis. Se & SF MRP 8/14 were determined by sandwich ELISA.

## Results

32 children (20 JIA, 12 DA) had Se samples taken for CRP, ESR & MRP 8/14 levels at diagnosis. 14 of these children had paired SF samples taken. Table 1 highlights accuracy of each measurement as a marker of disease activity. In DA, a significant correlation was detected between AJC & both ESR & MRP 8/14 (SF). Combining results for the DA & JIA cohort, a significant positive correlation was noted between paired samples of MRP 8/14 in Se & SF.

**Table 1:** 

*(n=32 serum, n=14 synovial fluid)*					
**Dx**	**Statistical test**	**Variable 1**	**Variable 2**	**Correlation**	**p value**

**DA**	***Correlation***	Active Joint	ESR	**Positive**	**p<0.05**
			
		Count	MRP 8/14 SF	**Positive**	**p<0.05**
			
		(AJC)	CRP	No Correlation	ns
		
		MRP 8/14 SF	ESR	**Positive**	**p<0.01**

**JIA/DA**	***Correlation***	MRP 8/14 SF	MRP 8/14 serum	**Positive**	**p<0.05**

**Dx**	**Statistical test**	**Variable 1**	**Variable 2**	**Outcome**	**p value**

**JIA/DA**	***Paired t test***	MRP 8/14 SF	MRP 8/14 Se	SF MRP8/14 signif.higher than matched SeMRP8/14	**p<0.01**

## Conclusion

MRI with contrast remains the gold standard for dx of synovitis. In reality, clinical assessment is the major diagnostic tool. DA is a more challenging condition than JIA, in light of confounding illness & the often-associated non-verbal state. In DA a simple biomarker of disease would be invaluable. We have shown that CRP is a poor marker of disease activity in JIA & DA, so the need for a more specific biomarker is evident. Our preliminary results suggest that children with DA have elevated SF levels of MRP 8/14 that correlate to disease activity. SF MRP 8/14 measurements are significantly higher than their paired Se samples, however our results show significant positive correlation between the two. This suggests that Se MRP 8/14 levels are potentially accurate markers of SF levels. MRP 8/14 may be a useful biomarker in DA, aiding timely dx & instigation of appropriate Rx, helping to improve clinical outcomes for this group.

## Disclosure of interest

None declared.

